# Supplementary material for: Antibiogram of clinical isolates from primary and secondary healthcare facilities: A step towards antimicrobial stewardship
Source: PLOS Glob Public Health. 2022 Dec 28;2(12):e0000644. doi: 10.1371/journal.pgph.0000644 (PMC10022303; doi:10.1371/journal.pgph.0000644)
Supplement: S1 Text — (DOCX) [file pgph.0000644.s001.docx]

**DATA COLLECTION TOOL: ASSESSING ANTIMICROBIAL SENSITIVITY & RESISTANCE PATTERN**

**Age:--------------------**

1. Year: (a) 2018 [ ] (b) 2019 [ ] (c) 2020 [ ] (d) 2021 [ ] 2. Sex: (a) male [ ] (b) female [ ]

3. Specimen: (a) urine [ ] (b) blood { ] (c) semen [ ] (d) Ear swab { ] (e) Wound swab [ ] (f) Vagina swab [ ] (g) other [ ]

4. Microorganism: (a) Ps aeruginosa [ ] (b) Coliform [ ] (c) S. aureus [ ] (d) MRSA { ] (e) E.coli { ] (f)

5. Study site: (a) Cen [ ] (b) UBTH [ ] [c] primary care center [ ]

6. Sensitivity profile:

Sensitive Resistant Sensitive Resistant

(a) Augmentin [ ] [ ] (b) Cefuroxime [ ] [ ]

(c) Ceftriaxone [ ] [ ] (d)ceftazidime [ ] [ ]

(e) Ofloxacin [ ] [ ] (f) Cefixime [ ] [ ]

(g) Ciprofloxacin [ ] [ ] (h) Erythromycin [ ] [ ]

(i)Nitrofurantoin [ ] [ ] (j) Gentamicin [ ] [ ]

(k) Ampicillin [ ] [ ] (L) Cloxacillin [ ] [ ]

(m)Amoxicillin [ ] [ ] (n)Levofloxacin [ ] [ ]

(o)Azithromycin [ ] [ ] (p) Cloxacillin [ ] [ ]

(q) Streptomycin [ ] [ ]
