# Supplementary material for: Antibiogram of clinical isolates from primary and secondary healthcare facilities: A step towards antimicrobial stewardship
Source: PLOS Glob Public Health. 2022 Dec 28;2(12):e0000644. doi: 10.1371/journal.pgph.0000644 (PMC10022303; doi:10.1371/journal.pgph.0000644)
Supplement: Attachment — Submitted filename: Response to Reviewers.docx [file pgph.0000644.s003.docx]

**PGPH-D-22-00846
Antibiogram of Clinical Isolates from Primary and Secondary Healthcare Facilities: A Step Towards Antimicrobial Stewardship
PLOS Global Public Health**

**Journal Requirements:**

1. In the Methods section and the online submission form, please provide additional information about the patient records used in your retrospective study. Specifically, please ensure that you have discussed whether all data were fully anonymized before you accessed them and/or whether the IRB or ethics committee waived the requirement for informed consent. If patients provided informed written consent to have data from their medical records used in research, please include this information.

**Response:** Details of the research objectives and methods regarding the data collection for thus retrospective study were clearly stated, including the fact that patient’s specific details such as name and contact details will not be obtained during application for ethical approval and administrative approval. For the ethics committee to have given approval for the study, the authors consider that the requirement for patient informed consent was waived. Moreso, staffs in the various facilities were monitoring the data collection process. Hence, the authors have added that requirement for patient informed consent was waived in the method and ethical approval section.

2. Please provide separate figure files in .tif or .eps format and remove the embedded figures within the manuscript file.

For more information about figure files please see our guidelines:

<https://journals.plos.org/globalpublichealth/s/figures> 
<https://journals.plos.org/globalpublichealth/s/figures#loc-file-requirements>

**Response:** The figure has been removed and added in a separate file following your Journal guideline

3. We have noticed that you have uploaded Supporting Information files, but you have not included a list of legends. Please add a full list of legends for your Supporting Information files after the references list.

**Response:** Thank you for noticing. We have included a list of legend for the Supporting Information Files.

Additional Editor Comments (if provided):

I have reviewed your manuscript alongside an additional reviewer, whose feedback can be found below.
This manuscript makes a valuable contribution on the characterisation and surveillance of rising AMR across the globe. Despite large scale surveillance studies in higher income countries, very little is known on the prevalence and distribution of AMR in LMICs.

**Response:** Thank you for this acknowledgement**.**

Specific points:
Highlight low numbers of samples.

**Response: this is true. Authors have added it as part of limitation of study as “**A significant limitation of this study is the low number of samples collated from the various facilities during the study period, this limitation is further seen with few isolates for some bacteria such as proteus, *Streptococci Pneumonia* and *Neisseria gonnorhea* below the required count of 30 isolates necessary to be used for culture and sensitivity tests”

Additional detail in the methods on patient recruitment, bacterial identification and antibiotic susceptibility testing.

**Response:** Additional details have been added to the revised version in the methods section

The results section needs more explanatory detail, highlighting the rationale and key findings of each analysis.

**Response:** More details has been added to the result section. Table 1 has been modified to accommodate more details, same was done to the text introducing the table. The text after Table one has been merged with the text before it for better organization. More explanatory details have been added to legends to tables 4 and 5.

Additional detail in figure legends.

**Response:** The text introducing the figures have been merged and additional details have been added to figure legends. In the course of this revision, the authors noticed some errors in figure 2, regarding resistance report for ceftriaxone 2019 (8% not 92% in the previous figure), azithromycin 2021 (76% not 81%), clindamycin 2021 (40% not 4%) and levofloxacin 2020 (36% not 26%) 2021 (32% not 78%). The authors are deeply sorry for the mix up, and have diligently scrutinized all details of the study in the revised manuscript. Thank you for pointing this.

Reviewer #1: The manuscript is really interesting and brings unique data on antibiograms and stewardship in LMICs. However it the limited samples should be highlighted as a limitation of the study. Urine, semen and vaginal swabs might not be the main reason of consultation in PHC and particularly not in SHC, it would be good to explain a bit more why these were the more common samples rather than blood, CSF or respiratory samples.

**Response:** Thank you for the time it took you to review our manuscript, and for finding it interesting. The issue of limited samples has been added to limitations of study. As mentioned, consultations to SHC and PHC are not limited to urine, semen and vaginal samples. From our study, these were the common samples requested for laboratory investigation over the study periods from both facilities. One reason for this observation is that respiratory samples and CSF are more associated with paediatrics patients and physicians in these facilities may tend to adopt presumptive diagnosis in treatment of these patients, and considering the fact that viruses cause respiratory infections more than bacteria. Moreso, investigation with CSF requires more expertise which may be lacking in primary care settings. Another possibility is that some patients might have treated infections associated with urinary tract and the vagina on their own from patent medicine dealers and pharmacy outlets most of which do not ask patients to conduct microbial test before treatment as is common in LMICs, hence recurrent infections warrant them to visit these facilities. This has been added to the discussion section.

The paper would benefit of making the link between the microbiology findings and the main syndromes/diagnosis seen at PHC and SCH; this would emphasize the need of similar studies to better define clinical guidelines for the more common syndromes (LRTI, BSI, Meningitis, etc). Very few studies show S aureus as a cause of UTI or vaginal/prostatic infections, and if this is the case in this region , it deserves a complete approach towards guidelines.

**Response:** The result section highlights the microbiology findings from SHC and PHC.

**“**Urine (51.8%), semen (16.5%), wound swab (13.3%) and vagina swab (6.9%) represented majority of the specimen in SHC, while urine (46.9%), vaginal swab (32.8%) and semen (14.6%) were most encountered in PHC. *S.aureus* (60.6%) was the microorganism most isolated while coliform organism (31.9%) represented about one-third of the microorganisms. *Ps aeruginosa* and proteus were isolated from SHC only. The only Gonorrhea isolate was seen from PHC while the PHC differentiated *E.coli* from other coliforms unlike in SHC where all are grouped together (See Table 1). About half (51.8%) of the isolates were collected from females with urine (54.7%), wound swab (12.3%) and vagina swab (25.5%) constituting most of the samples. In males, most samples were from urine (46.3%), semen (33.4%) and wound swab (8.8%). Coliform organisms and *S.aureus* constituted majority of the organisms isolated from all cultures with highest isolates of *S.aureus* from urine (45.7%), semen (21.7%), vagina swab (16.3%); for coliform, urine (44.4%) and wound swab (16.8%). *Ps Aeruginosa* was more isolated from wound swab (22.7%) and semen (27.2%). Most E. coli isolated was from urine (56.2%) and vagina swab (31.2%), all streptococci pneumonia was isolated from urine, proteus specie was isolated from urine (50%) and ear swab (50%) respectively, while the only *N. Gonorrhea* was found in vagina swab” **(excerpts from the result showing Microbiological analysis, hence, the inference that infections affecting the urinary tract and reproductive tract were most seen in our study as seen in the discussion section).**

**The last sentence of your comment has been added to recommendation based on our research findings for possible consideration in the discussion part.**

Lastly, a mention on bacteria intrinsically resistance to some antibiotics should be mention in order to avoid confusions. Some statements like high resistance to Azytromicine should be seen in the clinical context: none of the infections described (uro genital) will be treated with Azytromicine.

**Response:** Thank you for this insight. It is true that some bacteria such as *pseudomonas aeruginosa* and *staph aureus* exhibit intrinsic resistance to some B-lactam antibiotics and like you mentioned observed intrinsic resistance for azithromycin. Results from this study clearly show high resistance to other antibiotics too such as the floroquinolone class and aminoglycoside that have been shown to be very effective against some of the microorganisms isolated in our study. However, the results from the study are reflection of what is likely obtainable in clinical practice, especially as some clinicians in this region do not usually comply with recommendations and guidelines for antibiotic prescription. Moreso, antibiotics like azithromycin is widely used in addition to other drugs for treatment of sexually transmitted infections which can affect the urinary tract and he reproductive tract in the study area. Hence, the authors discuss findings in general terms both acquired and intrinsic resistance.
